# Piperacillin-Tazobactam Strikes Again: A Neutropenic Mystery Unraveled

**DOI:** 10.7759/cureus.93189

**Published:** 2025-09-25

**Authors:** Prabhu Ramalingam, Zeinab Nasser, Kunj Patel, Vrushali Dabak

**Affiliations:** 1 Internal Medicine, Baton Rouge General Medical Center, Baton Rouge, USA; 2 Hematology and Medical Oncology, Henry Ford Health System, Detroit, USA

**Keywords:** adverse drug events, bone marrow suppression, bone marrow toxicity, drug induced neutropenia, hematology and oncology, leukopenia, leukopenia - low white cell count, piperacillin, piperacillin-tazobactam induced agranulocytosis, piperacillin-tazobactam induced neutropenia

## Abstract

Piperacillin-tazobactam is a broad-spectrum antibiotic commonly used in inpatient settings, particularly for febrile neutropenia. While its known adverse effects are mainly gastrointestinal, rare hematologic effects have been reported. This case describes a patient who developed leukopenia and severe neutropenia after prolonged use of piperacillin-tazobactam. A male in his 60s was transferred to our facility for specialized care following Hartmann's procedure for an obstructing sigmoid mass. He was on piperacillin-tazobactam for suspected sepsis. After 24 days of therapy, he developed severe neutropenia without signs of infection or other underlying causes. Hematology evaluation ruled out alternative explanations, and piperacillin-tazobactam was identified as the probable cause. Upon discontinuation, his blood counts gradually improved without requiring granulocyte colony-stimulating factor (G-CSF) support. Leukopenia due to piperacillin-tazobactam is rare and likely dose- and duration-dependent, potentially caused by direct toxicity to myeloid precursors. Other hematologic effects like hemolytic anemia and thrombocytopenia have been documented. Prolonged use of piperacillin-tazobactam, especially in at-risk groups, can lead to hematologic toxicity. Clinicians should monitor blood counts during extended therapy and consider early discontinuation if adverse effects arise.

## Introduction

Piperacillin-tazobactam is a semi-synthetic aminobenzyl penicillin derivative used as a broad-spectrum parenteral antibiotic against predominantly Gram-negative and a few Gram-positive organisms in the inpatient setting. Piperacillin provides its antibiotic effect by binding to penicillin-binding proteins and inhibiting peptidoglycan synthesis in the bacterial cell wall, thereby leading to its bactericidal action [1]. It is often combined with tazobactam, a beta-lactamase inhibitor, to enhance the effect of beta-lactam antibiotics [1]. It is particularly effective in the treatment of infections caused by beta-lactamase-producing bacteria [2,3]. It is also one of the most common drugs used in the treatment of febrile neutropenia. The commonly known adverse effects of piperacillin-tazobactam are usually hypersensitivity reactions and gastrointestinal effects, such as diarrhea, increasing the risk for *Clostridium difficile* colitis, and other skin reactions like urticaria. They can rarely cause hematologic adverse effects secondary to bone marrow suppression, presenting as agranulocytosis, anemia, hemophagocytic lymphohistiocytosis, thrombocytopenia, and leukopenia. The incidence of hematological adverse effects after piperacillin-tazobactam is ≤1% for most effects in general populations, but neutropenia rates may be substantially higher, up to 34%, with prolonged or high-dose therapy, especially in children and patients with bone infections [4-6]. Here, we describe a case of piperacillin-induced reversible bone marrow suppression presenting as leukopenia and severe neutropenia in our inpatient setting.

## Case presentation

**Figure 1:**
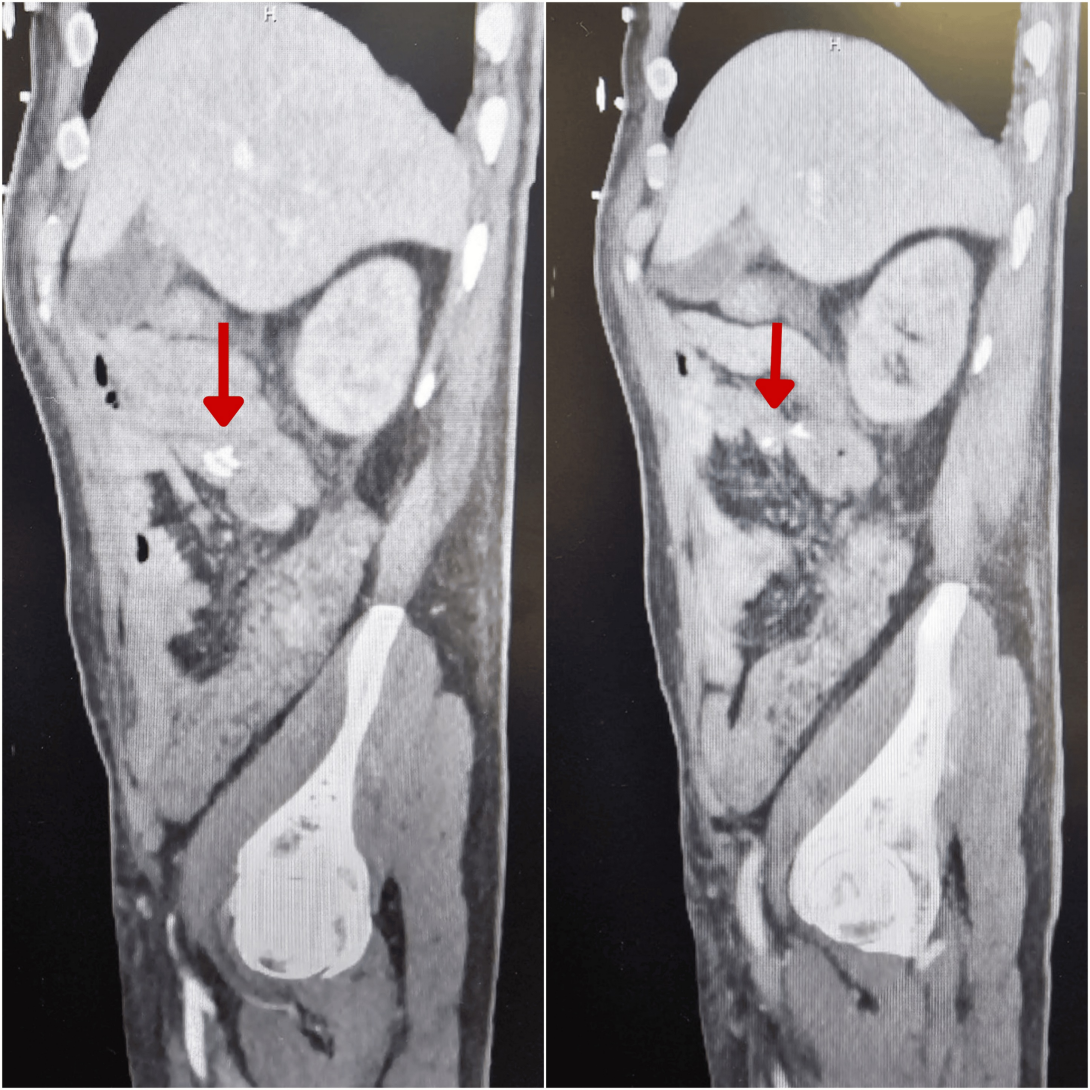
CT angiogram of the abdomen and pelvis showing a blush of intraluminal contrast adjacent to anastomotic structures in the right upper abdomen concerning for GI bleed (arrows).

**Figure 2:**
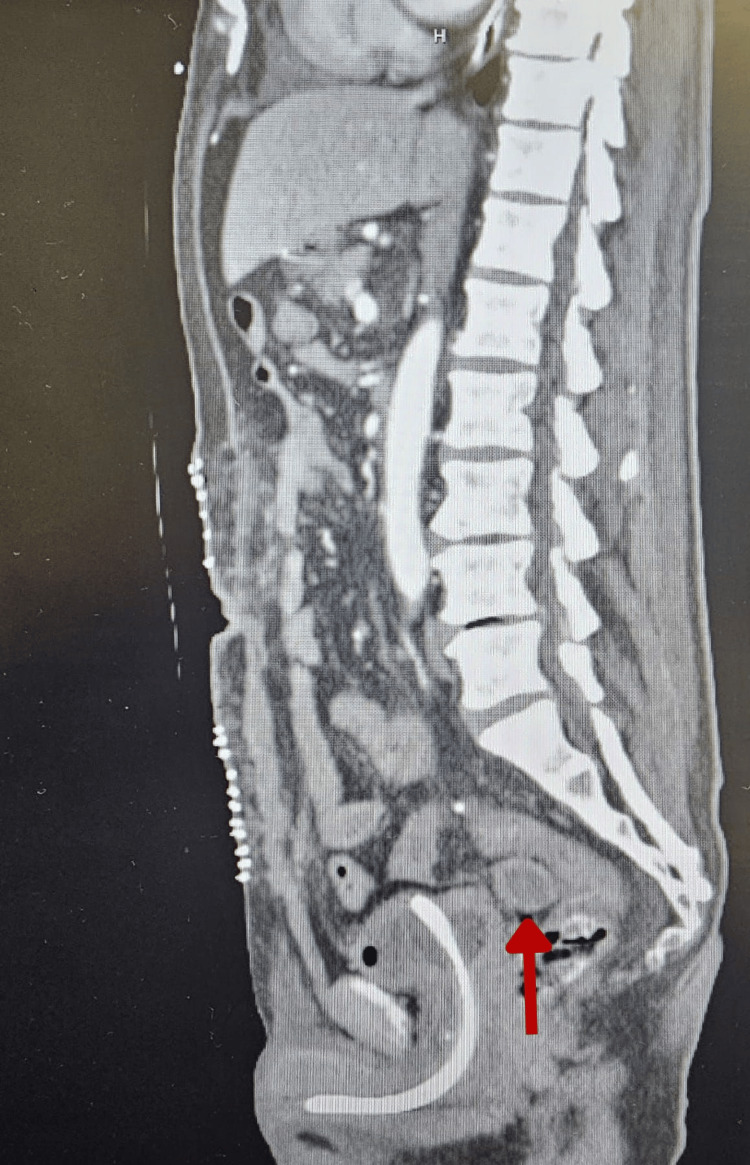
CT angiogram of the abdomen and pelvis describing a 3.5 cm fluid collection between the bladder and rectum (arrow).

This is a 69-year-old male patient with no past medical history who was transferred to our facility from a satellite center for higher-level specialty care. He initially presented to the other facility with a two-week history of progressively worsening abdominal pain, distension, and constipation. On arrival, a CT scan of the abdomen demonstrated minimal stool burden, multiple dilated loops of bowel with a transition point at the sigmoid colon, and a markedly distended, fluid-filled stomach and distal esophagus suggestive of an obstructive neoplasm or colonic stricture. An initial attempt at flexible sigmoidoscopy by the general surgery team was unsuccessful. The patient was initiated on intravenous broad-spectrum antibiotic therapy with piperacillin-tazobactam due to clinical signs concerning for early sepsis. He subsequently underwent an exploratory laparotomy, which revealed an obstructing sigmoid colon mass. Hartmann's procedure was performed. Intraoperatively, a jejunal perforation was also identified, necessitating segmental resection with primary end-to-end anastomosis. Operative documentation noted significant dilatation of both the small and large bowel. Despite attempts at decompression via enterotomy, colotomy, and drainage of fecal contents, bowel distension persisted, leading to temporary abdominal closure utilizing an AbThera negative pressure therapy system. A nasogastric tube was placed, and the patient was transferred to the intensive care unit postoperatively. His course was complicated by acute kidney injury requiring initiation of hemodialysis. Two days following the initial surgery, he was returned to the operating room, where evidence of bowel decompression was observed, and definitive closure of the fascia and skin was performed. In the postoperative period, he developed bloody output from the ostomy and a decline in hemoglobin levels without concurrent cytopenias. A CT angiogram (Figure 1) revealed a blush of intraluminal contrast near the ileal anastomotic suture line, suggestive of active bleeding. Interventional radiology (IR) performed embolization of a small ileal arterial branch supplying the anastomotic site, though the procedure was unsuccessful. Additionally, he also underwent CT-guided aspiration of an intra-abdominal fluid collection (Figure 2) identified on postoperative imaging, which was found to be noninfectious.

The patient continued to receive piperacillin-tazobactam and underwent multiple transfusions during his hospitalization, including 19 units of packed red blood cells, three units of fresh frozen plasma, and two units of platelets. The most recent transfusion occurred approximately two weeks prior to his transfer to our facility for escalation of care and consideration of double-balloon enteroscopy. Endoscopic evaluation through the colostomy revealed blood and clots throughout the colon and extending into the distal ileum (visualized up to 30-40 cm), though no active source of bleeding was identified. Capsule endoscopy also failed to localize the source of bleeding. Upon arrival at our facility, he was admitted to the hospital medicine service and continued on piperacillin-tazobactam (4.5 g every 12 hrs). Shortly after admission, a significant decline in his white blood cell count was noted on hospital day 24 of piperacillin-tazobactam therapy, progressing to severe neutropenia. Laboratory assessment, when evaluated by hematology with thyroid-stimulating hormone (TSH), vitamin B12, folate, and iron levels, returned within normal limits (Table 1).

**Table 1:** Laboratory investigations. TSH: thyroid-stimulating hormone; CRP: C-reactive protein

Laboratory Tests	Values
Vitamin B12	306 (271-1000) (pg/ml)
Folate	12.8 pg/ml (>5.4)
TSH	0.87 (mIU/L)
C4 levels	15 (10-43) (mg/dL)
C3 levels	136 (82-193) (mg/dL)
Ferritin	416 (14–338) (ng/ml)
CRP (at previous facility)	66.8 (1.0-10.0) (mg/L)
Acute hepatitis panel	Non-reactive
HIV	Non-reactive
Blood cultures	No growth
Intra-abdominal fluid aspirate	No growth

**Figure 3:**
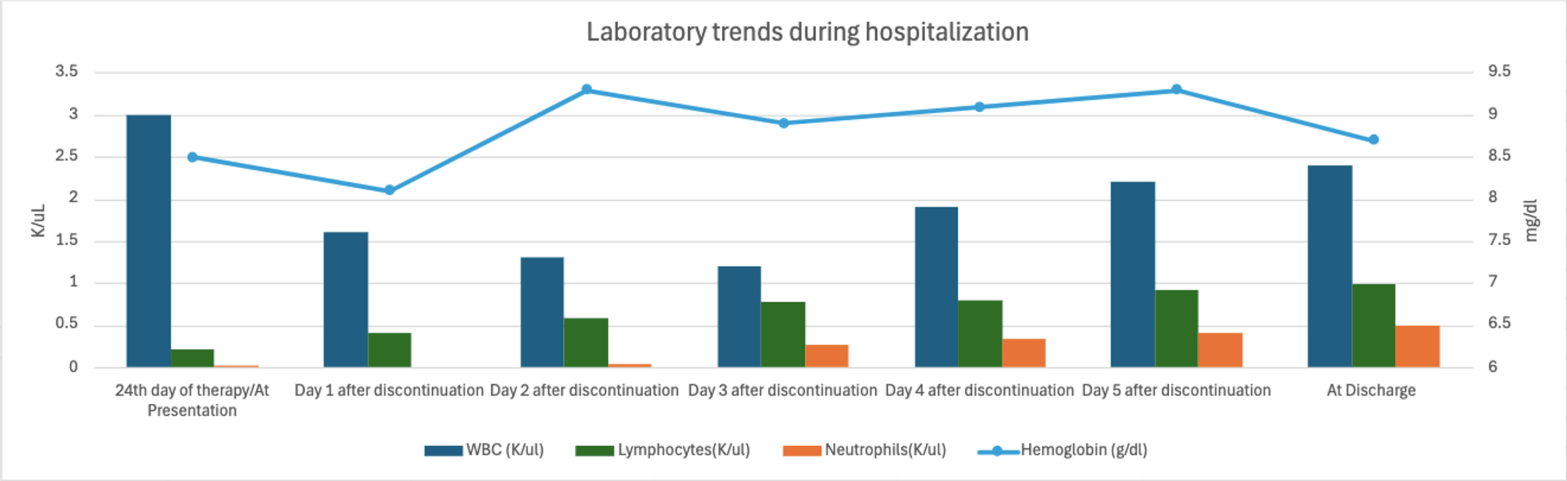
Pertinent laboratory trends during hospitalization.

He did not meet clinical criteria for sepsis or systemic inflammatory response syndrome (SIRS), making infection-related marrow suppression less likely. A peripheral smear demonstrated marked leukopenia with the absence of neutrophils, without evidence of schistocytes, dysplasia, or blasts. Given the onset of severe neutropenia after more than three weeks of antibiotic therapy, a comprehensive review of the patient’s medication regimen was undertaken to identify potential iatrogenic contributors. Piperacillin-tazobactam, which had been administered continuously since the initial presentation for presumed intra-abdominal infection, emerged as the most probable offending agent. As a rare adverse effect, prolonged use of piperacillin-tazobactam has been associated with reversible bone marrow suppression, causing neutropenia. After a multidisciplinary discussion with the Infectious Diseases team, a decision was made to discontinue piperacillin-tazobactam. Following cessation of the antibiotic, the patient’s absolute neutrophil count and overall white blood cell count began to improve steadily over subsequent days (Figure 3, Table 2). This temporal association strongly supports a diagnosis of piperacillin-tazobactam-induced transient bone marrow suppression as the underlying cause of the hematologic abnormalities.

**Table 2:** Important laboratory trends during hospitalization.

Parameters	24th day of therapy/at presentation	Day 1 after discontinuation	Day 2 after discontinuation	Day 3 after discontinuation	Day 4 after discontinuation	Day 5 after discontinuation	At discharge
WBC (K/ul)	3	1.6	1.3	1.2	1.9	2.2	2.4
Neutrophils(K/ul)	0.01	0	0.03	0.27	0.34	0.4	0.5
Lymphocytes(K/ul)	0.21	0.41	0.59	0.78	0.8	0.92	0.98
Hemoglobin (g/dl)	8.5	8.1	9.3	8.9	9.1	9.3	8.7

Differential diagnosis

The differential diagnosis for this patient's acute cytopenias primarily included bone marrow suppression secondary to sepsis, causing exhaustion of marrow granulocyte reserves, nutritional deficiencies, endocrinopathies such as severe hypothyroidism, and drug-induced myelosuppression. Although the patient experienced significant gastrointestinal bleeding, which contributed to anemia, his hemoglobin levels stabilized following appropriate surgical intervention and blood transfusions. Importantly, at the time of initial presentation, there were no significant abnormalities in his complete blood count, hemoglobin, white blood cell count, absolute neutrophil count, and platelet count, all within acceptable ranges. This makes primary hematologic malignancies such as myelodysplastic syndrome or myelofibrosis less likely.

Infectious etiologies were also explored. Testing for HIV and viral hepatitis returned negative, and he remained afebrile throughout his hospitalization at our facility. He demonstrated no signs of systemic inflammatory response or hemodynamic instability, effectively ruling out occult sepsis as a trigger for transient marrow suppression. Furthermore, the last transfusion occurred approximately two weeks prior to the onset of neutropenia. In the absence of pancytopenia or hemolytic laboratory findings, the probability of transfusion-related immune-mediated cytopenias, such as graft-versus-host disease or alloimmune neutropenia, is less likely.

A comprehensive nutritional and metabolic evaluation was also undertaken. Laboratory analysis of thyroid-stimulating hormone (TSH), vitamin B12, folate, and iron parameters returned within normal limits, thereby excluding nutritional or endocrine causes of cytopenia. Furthermore, complement levels were determined to be within the normal range, rendering any complement-mediated process a less probable etiology. With these etiologies ruled out, attention turned to potential medication-induced marrow suppression. Given that the patient had been receiving piperacillin-tazobactam continuously for over three weeks and the timing of the hematologic decline correlated directly with this prolonged antibiotic course, the drug was strongly suspected as the cause. The patient’s neutrophil count began to recover gradually over the subsequent days, establishing a temporal association supportive of piperacillin-tazobactam-induced bone marrow suppression.

## Discussion

Neutropenia is a very uncommon adverse effect of piperacillin-tazobactam secondary to an unclear mechanism. Several studies have suggested that piperacillin-tazobactam-induced neutropenia is more commonly associated with prolonged treatment duration and high cumulative dosing (144-480 g). Neutropenia rarely occurs within the first 10 days of therapy. Reported cumulative doses associated with neutropenia have ranged from 54 to 378 grams (mean: 259.6 grams), with treatment durations spanning five to 28 days (mean: 19.7 days) [1,5]. 

A retrospective cohort study in patients treated with piperacillin for osteomyelitis showed that 34% (14 of 41) of patients treated for more than 10 days developed neutropenia. It is of note that cumulative doses of piperacillin administered to neutropenic patients were higher (144-480 g) than those administered to non-neutropenic patients [6]. The hazards of high-dose, long-term β-lactam therapy for endocarditis have been highlighted by similar prospective research. The authors reported a clear dose-response relationship for adverse events. They concluded that benzylpenicillin at 16.9 g/day produced a high incidence of fever and rash. Furthermore, a new practice recommendation was suggested to limit daily intake to 12 g for therapeutic courses exceeding two weeks [7]. A retrospective review of 38 children with cystic fibrosis (mean age 14 years) undergoing treatment with piperacillin-tazobactam identified six cases presenting with fever, malaise, and headache in the absence of acute infection. Neutropenia developed in three patients between days 11 and 15 of therapy, all of whom had received higher cumulative doses of piperacillin-tazobactam compared to those without hematologic changes. Blood counts returned to normal following discontinuation of the drug [8,9]. 

The mechanism is possibly related to direct toxicity to myeloid precursors, causing proliferative arrest in the bone marrow in a dose-dependent fashion, which is usually reversible upon discontinuation of the drug. These findings support the association between extended piperacillin-tazobactam exposure and the risk of bone marrow suppression, consistent with the timeline observed in our patient (total cumulative dose received 216 g) [1,4,10,11]. In addition to the direct toxicity model, there is also evidence pointing towards an immunologically mediated mechanism in certain instances. A published case report documented immunologically mediated neutropenia, where the presence of detectable IgG antibodies against piperacillin was identified. This suggests that in some individuals, the body's immune system may mount a response, producing antibodies that specifically target and destroy neutrophils or their precursors following exposure to piperacillin, thereby resulting in neutropenia [12]. 

Piperacillin-induced hemolytic anemia (PIHA) was not identified during pre-approval clinical trials but has since been documented in post-marketing surveillance. While the exact incidence remains unknown, PIHA appears to be rare, with approximately 40 cases reported in the literature to date, including two associated fatalities. It is hypothesized that repeated exposure to piperacillin may increase the risk of PIHA by enabling multiple hapten-antigen interactions, thereby promoting immune-mediated hemolysis. Given this potential mechanism, clinicians should consider limiting repeated piperacillin use when possible and closely monitor patients receiving extended or recurrent courses of therapy with serial complete blood counts to allow for early detection of hematologic complications [11,13,14]. 

Piperacillin has been proposed to act as a hapten by binding to the platelet membrane and forming a drug-platelet complex, through which the synthesis of drug-dependent antibodies is induced. These antibodies are subsequently bound to platelet membrane glycoproteins, resulting in platelet consumption and thrombocytopenia [1]. Thrombocytopenia has also been reported as a rare adverse effect of piperacillin-tazobactam, potentially occurring through a non-immune-mediated mechanism [15]. In severe cases of drug-induced neutropenia, granulocyte colony-stimulating factor (G-CSF) may be considered as a supportive therapy. While multiple case reports and series have suggested that G-CSF can shorten the duration of neutropenia associated with non-chemotherapy medications, current evidence from prospective randomized studies has not demonstrated a significant clinical benefit [16]. In the case presented, the patient’s hematologic parameters improved following discontinuation of piperacillin-tazobactam alone, without the need for G-CSF. 

## Conclusions

This case report underscores the association between beta-lactam antibiotics, particularly piperacillin-tazobactam, and hematologic adverse effects. Although rare, complications such as neutropenia and thrombocytopenia can arise, particularly in patients with identifiable risk factors. These include prolonged duration of therapy, high cumulative antibiotic doses, low body weight, and impaired renal function, all of which may lead to drug accumulation and increased toxicity. Clinicians should maintain a high index of suspicion for such adverse effects and remain vigilant in monitoring patients receiving extended courses of these antibiotics. Early recognition and timely discontinuation or substitution with an alternative agent can result in complete hematologic recovery and prevent further complications. Regular monitoring of complete blood counts (CBCs), at least weekly CBCs, is strongly advised throughout the course of therapy, particularly in at-risk patients. Dose adjustments based on renal function are also critical to avoid drug accumulation and enhance patient safety. Judicious use, careful monitoring, and early intervention remain key strategies in minimizing the risk of hematologic toxicity from this widely used antibiotic. 
